# Mortality rates in Israel from causes amenable to health care, regional and international comparison

**DOI:** 10.1186/2045-4015-1-41

**Published:** 2012-10-25

**Authors:** Nehama Goldberger, Ziona Haklai

**Affiliations:** 1 Department of Health Information, Ministry of Health, 4 Shalom Yehuda Street, Jerusalem, 93480, Israel

**Keywords:** Amenable mortality, Regional differences, Causes of death, Periphery, Health services

## Abstract

**Background:**

Mortality from causes amenable to health care is a valuable indicator of quality of the health care system, which can be used to assess inter-regional differences and trends over time. This study investigates these mortality rates in Israel over time, and compares inter-regional and international rates in recent years.

**Results:**

Age-adjusted amenable mortality rates have been decreasing steadily in Israel, by 31% for males and 28% for females between 1998–2000 and 2007–2009. Amenable mortality was lower in the center of the country than in the Northern, Southern, and Haifa districts. The proportion of mortality from circulatory diseases was highest in the North and Haifa districts and from cancer in the Tel-Aviv and Central districts. A higher proportion of infectious diseases was seen in the Southern district.

In comparison with amenable mortality rates in 20 European countries, Israel ranked 8^th^ lowest for males and 12^th^ lowest for females, in 2008. The rate was lower than in Britain, Ireland, and Portugal; lower than in Germany, Spain, Austria, and Finland for males; and higher than France, Netherlands, Sweden, Norway, and Italy. But Israel ranked higher in the decrease in amenable mortality rates between 2001 and 2007 for females than males in a 19 country comparison. Genitourinary diseases were a larger component in Israel than other countries and circulatory diseases were smaller.

**Conclusion:**

The indicator of amenable mortality shows improvement in health outcomes over the years, but continuing improvement is needed in health care and education, in particular in the periphery of Israel and for females.

## Background

Indicators of health care performance are important to enable comparison between districts, between countries, and to assess changes over time. A good indicator should (1) reflect the overall performance of the health care system, (2) be objective, and (3) be based on accurate and available data. Mortality data fulfill the last two criteria, but since most deaths take place amongst the elderly, death rates may not completely reflect health care for the whole population. The concept of amenable mortality was developed and first used in the 1970s and 1990s, and has been the subject of recent research by Nolte and McKee [1,2] and Tobias and Yeh [3]. It is defined by Nolte and McKee [1] as “deaths from certain causes that should not occur in the presence of timely and effective health care.” A list of diseases are defined for which health interventions are deemed to exist that could prevent deaths before a certain age limit, with a general age limit of seventy-five years. Age-standardized mortality rates from these diseases before the age limit are calculated and used as an indicator. In a recent paper Gay et al. [4] reported on death rates from amenable causes in 31 OECD countries in 2007 or latest year available and their component causes, and compared the effect of differences in diseases chosen by Nolte and McKee and Tobias and Yeh. They found the choice of diseases in general made little difference to the ranking of countries, so in this study, we used the list developed by Nolte and McKee [1,2].

Amenable mortality rates were compared for different districts in Israel and over time from 1998 to 2009. International comparisons were made for 2007 and 2008, comparing only countries for which data was available for that year. Since amenable mortality is steadily decreasing in most countries, a correct international ranking can only be attained using data for the same year in all compared countries.

## Methods

The list of causes and age limits used is shown in Table 1. The main causes include deaths under age 75 from cancers of colon, rectum, skin, breast, cervix, uterus, and testis; leukemia; cerebrovascular disease; influenza, pneumonia, and other respiratory diseases; kidney disease; 50% of deaths from ischemic heart disease; deaths under age 50 from diabetes; and childhood deaths under age 15 from intestinal infections, whooping cough, and measles.

**Table 1:** **List of amenable causes of death**^**1**^

**ICD-10 codes**	**Age**	**Cause**
A00-9	0-14	Intestinal infections
A15-9, B90	0-74	Tuberculosis
A36, A35, A40-1, A80	0-74	Other infections (diphtheria, tetanus, septicemia, poliomyelitis)
A37	0-14	Whooping cough
B05	1-14	Measles
C18-C21	0-74	Malignant neoplasm of colon and rectum
C44	0-74	Malignant neoplasm of skin
C50	0-74	Malignant neoplasm of breast
C53	0-74	Malignant neoplasm of cervix uteri
C54,C55	0-44	Malignant neoplasm of corpus uteri and body of the uterus uterusuterusuterus
C62	0-74	Malignant neoplasm of testis
C81	0-74	Hodgkin’s disease
C91-C95	0-44	Leukemia
E00-E07	0-74	Diseases of the thyroid
E10-E14	0-49	Diabetes
G40-G41	0-74	Epilepsy
I05-I09	0-74	Chronic rheumatic heart disease
I10-I13, I15	0-74	Hypertensive disease
I20-I25	0-74	Ischemic heart disease: 50% of deaths
I60-I69	0-74	Cerebrovascular disease
J00-J09, J20-J99	1-74	All respiratory diseases (excl. pneumonia, influenza)
J10-J11	0-74	Influenza
J12-J18	0-74	Pneumonia
K25-K27	0-74	Peptic ulcer
K35-K38	0-74	Appendicitis
K40-K46	0-74	Abdominal hernia
K80-K81	0-74	Cholelithiasis and cholecystitis
N00-N07, N17-N19, N25-N27	0-74	Nephritis and nephrosis
N40	0-74	Benign prostatic hyperplasia
Y60-Y69, Y83-Y84	0-74	Misadventures to patients during surgical and medical care
O00-O99	0-74	Maternal death
Q20-Q28	0-74	Congenital cardiovascular anomalies
P00-P96	0-74	Perinatal deaths, all causes (excl. stillbirths)

^1^ From Nolte and McKee [1].

Israeli mortality data were taken from the nationwide database of causes of death prepared by the Central Bureau of Statistics (CBS) for the years 1998–2009, with underlying cause of death coded according to ICD-10.

International data were taken from the WHO Mortality database 2011 [4], which include cause and age-specific numbers of deaths and population estimates by year and country.

### Statistical analysis

Age-adjusted mortality rates were calculated by the direct method for total deaths due to causes amenable to health care. As in Gay et al. [5], the total OECD 2005 population was chosen for standardization for regional comparison and trends over time to allow comparisons between genders and gender-specific OECD 2005 population for international comparisons by gender.

## Results

### Amenable mortality in Israel, 1998–2009

**Figure 1:**
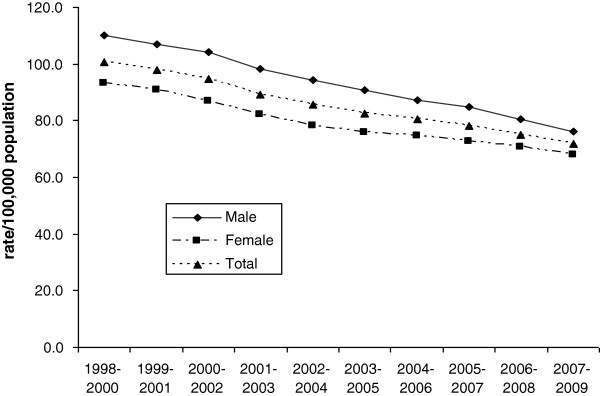
**Amenable mortality rates in Israel, 1998–2009.** Three-year-moving average rates per 100,000 persons of mortality amenable to health care, age-adjusted to total 2005 OECD population.

Figure 1 shows age-adjusted three-year moving average amenable mortality rates from 1998 to 2009 for Israel. Amenable mortality rates have been decreasing steadily, for males by 31% from 110.0 per 100,000 persons in 1998–2000 to 76.3 in 2007–2009, and for females by 28%, from 93.3 to 67.9, respectively. This is a greater decrease than that for total mortality rates for the corresponding period, similarly age adjusted, which decreased by 25% for males and 24% for females for deaths under age 75.

### Regional comparison, 2007–2009

**Figure 2:**
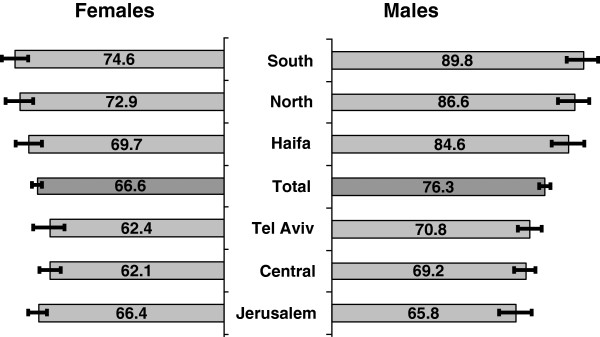
**Amenable mortality rates by district, 2007–2009.** Rates per 100,000 persons with 95% CI error bars, age-adjusted to total 2005 OECD population, ordered by male rates.

Figure 2 shows age-adjusted amenable mortality rates by district for the years 2007–2009. Rates were significantly higher for males in the Southern (89.8, 95% CI 84.1–95.6), Northern (86.6, 95% CI 81.0–92.3), and Haifa districts (84.6, 95% CI 78.9–90.3), 18%, 14%, and 11% greater than total country rate (76.3, 95% CI 74.3–78.3), respectively. For females, too, the rate in the Southern district (74.6, 95% CI 69.7–79.4) was significantly higher, 14% greater than the total country rate (66.6, 95% CI 64.9–68.3).

Rates were significantly lower for males in the Jerusalem (65.8, 95% CI 59.9–71.7) and Central districts (69.2, 95% CI 65.4–73.1), 14% and 9% less than the total country rate (76.3, 95% CI 74.3–78.3), respectively. For females, Central district rates (62.1, 95% CI 58.7–65.5) were lower than total country rate (66.6, 95% CI 64.9–68.3) although the difference was not significant, while Jerusalem rates were no lower than total country rate.

**Figure 3:**
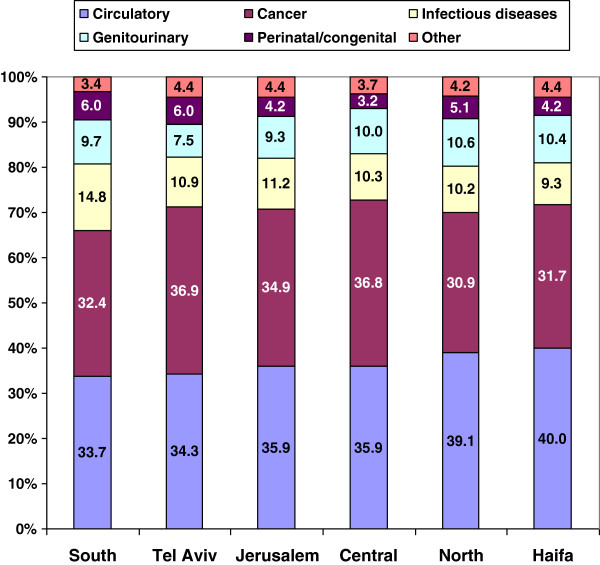
**Contributory causes to amenable mortality by district, 2007–2009.** Percentage of cause specific rates from total rate, all rates age-adjusted to total 2005 OECD population.

Figure 3 shows the percentage contribution of causes to total amenable mortality by district. Circulatory diseases contributed more and cancer less in the Northern and Haifa districts, while cancer contributed more in the Tel-Aviv and Central districts. The contribution of infectious diseases was particularly high in the Southern district, and genitourinary diseases contributed less in Tel-Aviv than other districts. Perinatal mortality contributed more in the Southern, Tel-Aviv, and Northern districts and less in the Central district. Similar patterns were found in the gender-specific breakdown.

### International comparison, 2008 and 2007

**Figure 4:**
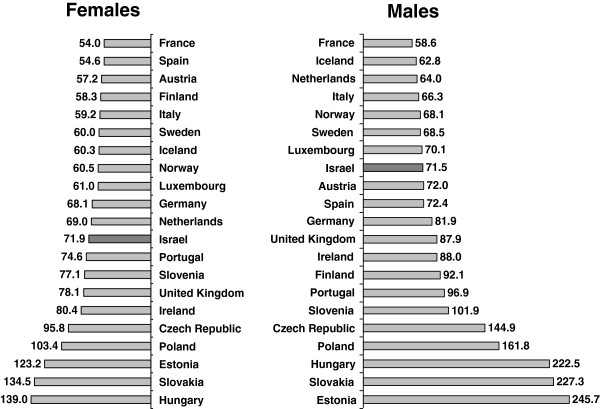
**International comparison of amenable mortality rates by gender, 2008.** Rates per 100,000 persons, age-adjusted to gender specific 2005 OECD population.

**Figure 5:**
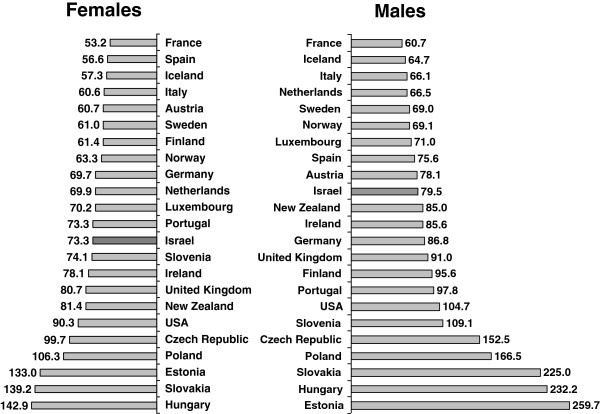
**International comparison of amenable mortality rates by gender, 2007.** Rates per 100,000 persons, age-adjusted to gender specific 2005 OECD population.

Figure 4 shows a comparison with 21 European countries for which data were available in 2008. Israel had the 8^th^ lowest rates of amenable mortality for males, and the 12^th^ lowest rates for females. Amongst Western European countries with higher rates than Israel were Britain, Ireland, and Portugal; for males higher rates were also seen in Germany, Spain, Austria, and Finland. Amongst Western European countries with lower rates than Israel were France, the Netherlands, Sweden, Norway, and Italy. Figure 5 shows a similar comparison with 23 countries for 2007, for which data were also available for the USA and New Zealand. Rankings were similar; Israel had the 10^th^ lowest rates for males, and 13^th^ lowest rates for females, and lower rates than the USA and New Zealand for males and females.

### International comparison, contributory causes in 2008

**Figure 6:**
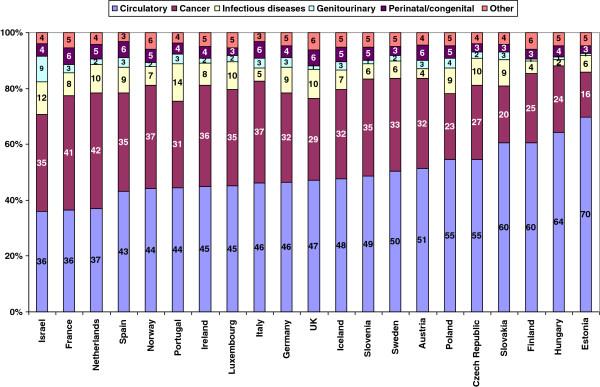
**Contributory causes to amenable mortality by country, 2008.** Percentage of cause-specific rates from total rate, all rates age-adjusted to total 2005 OECD population.

The contribution of different causes to total amenable mortality is shown for different European countries in 2008 in Figure 6. It is immediately apparent that Israel, along with France and the Netherlands, has the lowest contribution from circulatory diseases. The contribution from cancer is similar to that in Spain, Ireland, Norway, Luxembourg, and Sweden but lower than France and the Netherlands. Both infectious diseases and in particular genitourinary diseases contribute higher percentages than in other European countries.

### International comparison, decrease between 2001 and 2007

**Figure 7:**
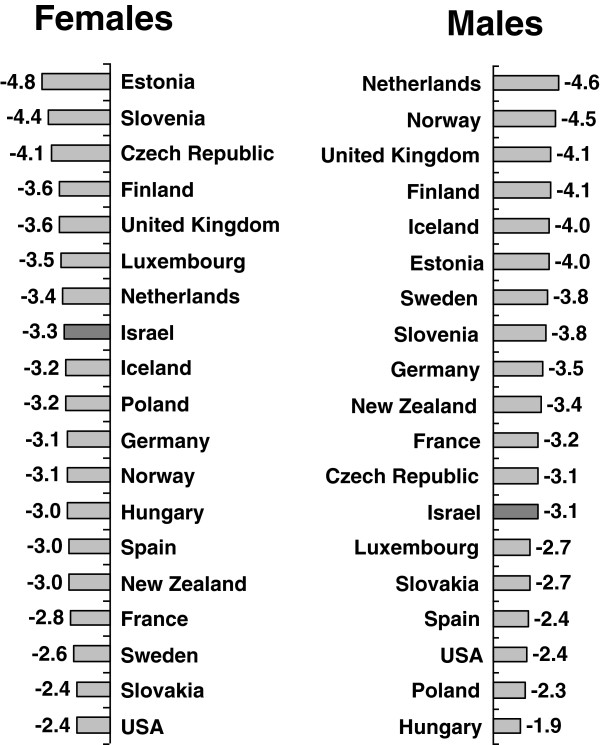
**International comparison of average annual percentage decrease in amenable mortality rates by gender, 2001–2007.** Rates per 100,000 persons, age-adjusted to gender specific 2005 OECD population.

The average annual decrease in age and gender age-adjusted amenable mortality between 2001 and 2007 is shown for 19 countries with data available for these years in Figure 7. Here, Israel ranks better for females, with the 7^th^ highest decrease of 3.3%, lower than Finland, the United Kingdom, and the Netherlands, but higher than Germany, Spain, Iceland, Norway, France, and the USA. For males, a similar decrease of 3.1% ranked 13^th^, lower than the Netherlands, Norway, the United Kingdom, Finland, Sweden, Germany, New Zealand, France, and the Czech Republic; and higher than Luxembourg, Spain, and the USA.

## Discussion

Amenable mortality has been proposed as a measure to assess health status changes and compare regional health outcomes. Gay et al. [5] discuss its value as an indicator of health system performance. They note that it is also influenced by prevalence of diseases, and not only success in treating them.

Nevertheless, the steady decrease in amenable mortality over time, and which was greater than the decrease in total mortality for these ages, would indicate success in treating those diseases considered amenable to health care. In particular, the Ministry of Health publication on causes of deaths [6] shows steep declines in death rates from heart and cerebrovascular disease at ages of amenable mortality, 1–74, between 1999–2001 and 2007–2009.

The decline was greater for males than females, but male rates remain higher than female rates, since amenable mortality includes under age 75 deaths only, and male mortality rates are considerably higher for those ages [6].

Significant regional differences were found, in particular among males, maybe because of their higher rates overall. The rates were particularly high in the peripheral Southern and Northern districts. We know from Ministry of Health data that the acute care inpatient beds rate is lowest for these districts, 1.38 and 1.48 per 1000 persons in the Southern and Northern districts in 2009 compared to 2.62, 2.50, and 2.23 in Haifa, Tel-Aviv, and Jerusalem, respectively [7]. The differences in rates of rehabilitation beds are even higher [7]. Similarly, the rate of employed doctors is lower, 1.6 and 2.2 per 1000 persons in the Northern and Southern districts in 2008–2010, compared to 5.2, 4.3, 3.8, and 3.8 in the Tel-Aviv, Haifa, Central, and Jerusalem districts [8]. Also, the rate of employed nurses is lower, 3.7 and 3.2 per 1000 persons in the Northern and Southern districts in 2008–2010, compared to 6.4, 6.0, 5.1, and 4.9 in the Haifa, Tel-Aviv, Central, and Jerusalem districts [8]. These may be contributory factors to the higher amenable mortality rates there. However, we see that Haifa which has a high rate of doctors and hospital beds, nevertheless has a higher amenable mortality rate than the country total, showing that other factors, such as disease prevalence discussed below, may also have a strong effect.

Amir Shmueli [9] has shown that doctors tend to be less available in poorer and less healthy localities, in particular more expensive specialists, such as internal medicine doctors and surgeons, due to economic considerations of the Heath Funds that provide medical services in the community. Despite the national health insurance law, weaker and less healthy populations have lower access to and availability of medical services, which leads to worse medical outcomes.

However, as Amir Shmueli has written in a recent article [10], the change in capitation payments to the Health funds in November 2010, which adds a periphery index to the formula for calculating the payment, giving monetary incentives to encourage Health Funds to provide services in the periphery, should improve services there. Similarly, the recent agreement with the doctors gives monetary incentives to those working in the periphery. It is to be hoped that these measures might redress some of the inequality in medical services reflected by our data. It remains important, too, to add inpatient and rehabilitation beds to the peripheral regions, and possibly give more economic incentives to Health Funds that demonstrate improvement in health care indicators in the periphery.

When we looked at the breakdown of amenable mortality by component causes, we also saw regional differences. These differences may well reflect the different prevalence of disease, too, due to differences in population groups, education, socio-economic level, and possibly environmental factors. For example, the Northern district population is composed of over 50% of Arabs and the Haifa district has 24% compared to 8% and 2% in the Central and Tel-Aviv districts, respectively [11], 2008. Age-adjusted mortality rates for circulatory diseases, and heart and cerebrovascular diseases are much higher for Arabs than Jews, particularly females [11], which may explain the larger proportion of these diseases in the Northern and Haifa districts. The high proportion of cancer in the Tel-Aviv and Central districts may similarly be due to the largely non-Arab population there who have higher mortality rates for many cancers than Arabs, but may also be connected to the higher socio-economic level of the population, or environmental factors. Cancer screening initiatives, such as encour aging mammography and screening for colon cancer, which allow early detection and treatment of these cancers, are particularly important in these areas. The high component of infectious diseases in the Southern district is of concern, and may be connected to the above mentioned lower availability of medical services and high number of Bedouins with poor access to sanitation services. Programs are needed to improve their conditions.

International comparisons show Israel in a relatively good position, particularly for males, as in 2008 Israel ranked 8^th^ lowest among 21 European countries for males, but 12^th^ for females. But Israel’s ranking in amenable mortality rates is not as good as for overall mortality rates, for which in 2009, male rates were the lowest in Europe, and female rates were amongst the lowest [6]. The decrease in amenable mortality in Israel over time is less than that in many other Western countries.

When we looked at the cause composition of the amenable mortality, we saw the low contribution of circulatory diseases, and relatively high contributions of genitourinary and infectious diseases. Similarly high rankings for kidney and infectious diseases (septicemia and flu/pneumonia) and low ranking for circulatory diseases (heart and cerebrovascular diseases) are reported by the Ministry of Health for total mortality [6]. The accuracy of all data on mortality causes depends on the accuracy with which causes of death are coded, which is turn also depends on how well doctors fill in the death certificates. The CBS in Israel codes according to international standards and has had quality control tests on the coding for over 10 years, with good results. But it is still possible that doctors tend to report certain diseases more than others. In an unpublished analysis of multiple causes of death that have been coded for the last few years, we found that about 40% of cases coded with kidney disease as an underlying cause, also mentioned a circulatory disease on the death certificate.

Nevertheless, the high proportion of the genitourinary component of amenable mortality in particular, and relatively high ranking of Israel compared to its low ranking for total mortality, could reflect unhealthy behavior of the younger population in Israel. This should be addressed by health education programs teaching healthy drinking and eating habits, such as avoiding too much salty fast food, encouraging exercise, discouraging smoking, and encouraging drinking more water, very important in Israel as a country with a hot climate. The Ministry of Health has begun initiatives in this direction but must continue and increase them.

Female amenable mortality for Israel ranks lower than that for males, and has been decreasing less rapidly. Amenable mortality rates are still lower for females than males, but their health awareness must be encouraged, and in particular, amongst Arab females, who have much higher mortality rates for many diseases [11].

## Conclusion

The indicator of amenable mortality shows improvement in health outcomes over the years, but a need for continuing progress in improving health care and education, in particular in the periphery of Israel and for women.

## Endnotes

Rates for the Jewish population of Judea and Samaria were also lower, although since this population has a much younger age distribution, the age adjustment does not adequately compensate for population differences, thus not allowing a true comparison with other regions, and therefore we did not present their data. Their low rates also reflect their relatively high socioeconomic and education level and their mobility.

## Competing interests

The authors declare that they have no competing interests.

## Authors’ contributions

NG analyzed the data and drafted the paper. ZH directed the analysis and edited the paper. All authors read and approved the final manuscript.

## Authors’ information

Nehama F Goldberger is a researcher in health statistics at the Health Information Division of the Ministry of Health.

Ziona Haklai is Director of the Health Information Division of the Ministry of Health.

## References

[B1] Nolte E McKee M Does Healthcare Save Lives? Avoidable Mortality Revisited 2004 London: Nuffield Trust

[B2] Nolte E McKee M Measuring the health of nations: updating an earlier analysis Health Affairs 2008 27 1 58 71 10.1377/hlthaff.27.1.58 18180480

[B3] Tobias M Yeh L How much does health care contribute to health gain and to health inequality? Trends in amenable mortality in New Zealand 1981–2004 Aust N Z Public Health 2009 33 70 78 10.1111/j.1753-6405.2009.00342.x 19236363

[B4] WHO Statistical Information System (WHOSIS) Detailed data files of the WHO Mortality Databasehttp://www.who.int/whosis/mort/download/en/index.html

[B5] Gay JG Paris V Devaux M de Looper M Mortality Amenable to Health Care in 31 OECD Countries: Estimates and Methodological Issues OECD Health Working Papers 2011 55 10.1787/5kgj35f9f8s2-en

[B6] Goldberger N Aburbeh M Haklai Z Leading causes of death in Israel, 2000–2009 Ministry of Health 2012 Hebrew http://www.old.health.gov.il/pages/default.asp?maincat=2&catid=841&pageid=4433

[B7] Inpatient institutions and Day Care Units in Israel 2010, part 1: Hospitalization Trends Health Information Division, Ministry of Health 2011 Hebrew http://www.old.health.gov.il/pages/default.asp?maincat=2&catid=514&pageid=5295

[B8] The Medical Workforce 2010 Health Information Division, Ministry of Health 2011 Hebrew http://www.old.health.gov.il/pages/default.asp?maincat=2&catid=667&pageid=5278

[B9] Shmueli A Engelson-Nissan E The inverse relation between the availability of doctors in the community and health requirements in Israeli localities: a failure in the capitation formula allocating the health resources to the Health funds? Presented at the 8th conference of the NIHP, Tel-Aviv, December 2008 [Hebrew]

[B10] Shmueli A Engelson-Nissan E What determines the market share of the Health funds in Israeli localities Harefuah 2011 150 8 650 654 688 Hebrew 21939116

[B11] Health in Israel 2010 Health Information Division, Ministry of Health 2011 Hebrew http://www.old.health.gov.il/pages/default.asp?maincat=2&catId=653&PageId=3607

